# Generation of Dopamine Neurons with Improved Cell Survival and Phenotype Maintenance Using a Degradation-Resistant Nurr1 Mutant

**DOI:** 10.1002/stem.146

**Published:** 2009-09

**Authors:** A-Young Jo, Mi-Young Kim, Hyun-Seob Lee, Yong-Hee Rhee, Jeong-Eun Lee, Kwang-Hyun Baek, Chang-Hwan Park, Hyun-Chul Koh, Incheol Shin, Yong-Sung Lee, Sang-Hun Lee

**Affiliations:** a Department of Biochemistry and Molecular Biology, College of Medicine, Hanyang UniversitySeoul, Korea; b Institute of Mental Health, College of Medicine, Hanyang UniversitySeoul, Korea; c Cell Therapy Research Center, College of Medicine, Hanyang UniversitySeoul, Korea; d Department of Pharmacology, College of Medicine, Hanyang UniversitySeoul, Korea; e Laboratory of Molecular Signal Transduction, Department of Biomedical Science, CHA University, CHA General HospitalSeoul, Korea; f Department of Microbiology, College of Medicine, Hanyang UniversitySeoul, Korea; g Department of Life Science, College of Natural Sciences, Hanyang UniversitySeoul, Korea

**Keywords:** Nurr1, Dopamine neuron, Ubiquitin-proteasome-system, Cell survival, Midbrain, Akt

## Abstract

Nurr1 is a transcription factor specific for the development and maintenance of the midbrain dopamine (DA) neurons. Exogenous Nurr1 in neural precursor (NP) cells induces the differentiation of DA neurons in vitro that are capable of reversing motor dysfunctions in a rodent model for Parkinson disease. The promise of this therapeutic approach, however, is unclear due to poor cell survival and phenotype loss of DA cells after transplantation. We herein demonstrate that Nurr1 proteins undergo ubiquitin-proteasome-system-mediated degradation in differentiating NP cells. The degradation process is activated by a direct Akt-mediated phosphorylation of Nurr1 proteins and can be prevented by abolishing the Akt-target sequence in Nurr1 (Nurr1^Akt^). Overexpression of Nurr1^Akt^ in NP cells yielded DA neurons in which Nurr1 protein levels were maintained for prolonged periods. The sustained Nurr1 expression endowed the Nurr1^Akt^-induced DA neurons with resistance to toxic stimuli, enhanced survival, and sustained DA phenotypes in vitro and in vivo after transplantation.

## INTRODUCTION

Midbrain dopamine (DA) neurons play essential roles in the control of voluntary movement and the regulation of emotion. Degeneration/dysfunction of this neuronal subtype underlies clinical features of many neurological and psychiatric disorders. Nurr1 (NR4A2), a transcription factor belonging to the orphan nuclear receptor family, is expressed in the developing midbrain and is critical for midbrain DA neuron development [1,2]. Nurr1 is also expressed in the DA neurons of the adult midbrain, and sustained expression of this factor has been reported to be crucial for the maintenance of dopaminergic phenotypes [1,3] and survival [2,4,5]. Reduced levels and genetic alterations of Nurr1 in adult midbrain DA neurons have been found in midbrain DA pathologies [6,7], indicating that a potential therapeutic strategy could be established through manipulation of Nurr1 protein level and function in patients with those disorders.

Neural precursor (NP) cells can be isolated from developing and adult brains, and cultured for the purpose of generating large numbers of donor cells to treat neurodegenerative disorders. Interest in Nurr1 has intensified due to its in vitro role in DA neuron generation from cultured NP cells. Exogenous Nurr1 expression in the absence [8] or presence of neurogenic factor coexpressions drives naïve nondopaminergic NP cells to differentiate into DA neurons that exhibit presynaptic functionalities capable of reversing dopaminergic deficits in a rodent model of Parkinson disease (PD). However, poor cell survival [8] and loss of DA phenotype of donor cells [9] after transplantation are the most critical concerns in these procedures.

The proteasomal degradation system is a critical regulator of protein activity in a cell, with various cellular proteins targeted to the proteasome for degradation by the covalent addition of multiple molecules of ubiquitin, a 76-amino acid polypeptide. In this report, we demonstrate that Nurr1 proteins undergo ubiquitin-proteasome-system (UPS)-mediated degradation in differentiating NP cells. Intracellular signals responsible for the protein degradation were defined and this molecular understanding of the degradation process led us to generate a ubiquitylation-resistant Nurr1 mutant. Induction of mutant protein expression in NP cells yielded DA neurons with Nurr1 protein levels that were stably maintained for a prolonged period while preserving native Nurr1 functions. As a consequence, DA neurons generated by the mutant Nurr1 were resistant to toxic stimuli and exhibited enhanced cell survival in vitro and in vivo in rat brains after transplantation. These findings represent a substantial technical advance in stem/precursor cell-derived DA neuron generation for PD cell therapy and provide important cues for developing strategies to prevent PD progression.

## MATERIALS AND METHODS

### Primary Culture for Neural Precursor Cells

Brain tissue was dissected from rat (Sprague Dawley) embryonic cortices at embryonic day 13. Dissected cortices were mechanically triturated in Ca^2+^/Mg^2+^ free Hanks' balanced salt solution (HBSS; Gibco, Grand Island, NY, http://www.invitrogen.com), seeded at 19,000 cells/cm^2^ on 10-cm culture dishes (Corning Life Sciences, Acton, MA, http://www.corning.com/lifesciences) precoated with polyornithine/fibronectin, and cultured for 4–5 days in serum-free N2 medium supplemented with basic fibroblast growth factor (bFGF; 20 ng/ml; R&D Systems Inc., Minneapolis, http://www.rndsystems.com). Cell clusters generated by precursor cell proliferation were dissociated in HBSS and plated at 50,000 cells/cm^2^ on coated 24-well and 6-well plates. After additional induction of precursor cell proliferation in N2+bFGF up to 60%–80% cell confluency (typically 1–2 days after plating), cells were subjected to retroviral transduction as described below. On the day following transduction, cell differentiation was induced by withdrawing bFGF for 4–12 days. The medium was changed every other day, and bFGF was supplemented daily. In certain experiments, the precursor cells were cultured in the form of floating cell aggregates (neurospheres) by seeding them on uncoated surfaces in the media supplemented with bFGF or epidermal growth factor (EGF; 20 ng/ml; R&D System). The following factors or inhibitors were used: FGF20 (20 ng/ml), NT3 (10 ng/ml), and BDNF (10 ng/ml; all from ProSpec-Tany TechnoGene, Rehovot, Israel, http://www.prospecbio.com/), MG132 (1–10 μM), and lactacystin (1–10 μM), SU5402 (20 μM), PD98059 (50 μM), U0126 (10 μM), LY294002 (20 μM), wortmannin (1 μM; from Calbiochem, San Diego, http://www.emdbiosciences.com) and leptomycin B (10 ng/ml; Sigma-Genosys, Cambridge, U.K., http://www.sigmaaldrich.com/Brands/Sigma_Genosys.html). Cell cultures were maintained at 37°C in a 5% CO_2_ incubator.

### Retroviral Construction and Infection

Retroviral vectors expressing Flag-tagged wild-type Nurr1 (Nurr1^WT^), Nurr1 mutant (Nurr1^Akt^), dominant negative form of Raf (dn-raf; kindly provided by Dr. Kang-Yell Choi, Yonsei University, Seoul, Korea), Wnt5a, and Notch intracellular domain (kindly provided by Dr. Jaesang Kim, Ewha Woman University, Seoul, Korea) were constructed by inserting each cDNA fragment into the multicloning sites of pCL [10]. Viral particles were produced by transfecting the retrovirus packaging cell line 293gpg with each vector using Lipofectamine (Invitrogen) and supernatants containing viral particles were harvested 72 hours after incubation. For viral transduction, prepared NP cells were incubated with the viral soup (5 × 10^6^ particles/ml) containing polybrene (1 μg/ml; Sigma-Genosys) for 2 hours, followed by a medium change with bFGF-supplemented N2. Coexpression studies were carried out by infecting cells with mixtures of the individual viral constructs (1:1).

### Immunofluorescent Staining

Cultured cells and brain tissues were fixed with 4% paraformaldehyde, blocked in 0.1% bovine serum albumin (BSA)/10% goat serum/0.3% Triton X-100 and incubated with primary antibodies overnight at 4°C. For detecting Nurr1-expressing cells grafted in brain sections, an antigen retrieval procedure was applied by treating cells with sodium dodecyl sulfate (1% in phosphate-buffered saline [PBS]) at room temperature for 5 minutes before the blocking procedure. The following primary antibodies were used: Nurr1 (1:200, Chemicon, Temecula, CA, http://www.chemicon.com, for cultured cells or 1:500, E-20, Santa Cruz Biotechnology Inc., Santa Cruz, CA, http://www.scbt.com, for detecting grafted cells in tissue), and tyrosine hydroxylase (TH; 1:250, Pel-Freez, Rogers, AK, http://www.invitrogen.com). Alexa 488- (1:200, Invitrogen) and Cy3- (1:200, Jackson Immunoresearch Laboratories, West Grove, PA, http://www.jacksonimmuno.com) labeled secondary antibodies were applied and mounted in Vectashield containing 4, 6-diamidino-2-phenylindole (DAPI, Vector Laboratories, Burlingame, CA, http://www.vectorlabs.com). Immunoreactive cells were analyzed under an epifluorescence microscope (Nikon Instruments, Melville, NY, http://www.nikoninstruments.com) or confocal microscope (Leica, Heerbrugg, Switzerland, http://www.leica.com).

### Western Blot and Immunoprecipitation Assays

Proteins were extracted from cultures, electrophoresed by sodium dodecyl sulfate polyacrylamide gel electrophoresis, and transferred to a nitrocellulose membrane. Transferred proteins were blocked in 5% nonfat milk in 0.001% Tween 20 with Tris Buffered Saline. Working concentrations of primary antibodies were as follows: Nurr1 (1:1,000, Chemicon), TH (1:1,000, Pel-Freez), β-galactosidase (β-gal, 1:1,000, MP Biomedicals, Irvine, CA, http://www.mpbio.com), extracellular regulated kinase (ERK; 1:1,000), phosphorylated extracellular regulated kinase (pERK, 1:1,000), Akt (1:1000), phosphorylated Akt (pAkt, Ser473, 1:1,000, Cell Signaling Technology, Beverly, MA, http://www.cellsignal.com), hemagglutinin (HA) (1:1,000, Covance, Princeton, NJ, http://www.covance.com), pAkt substrate (1:1,000, Sigma-Genosys), and β-actin (1:5,000, Abcam, Cambridge, U.K., http://www.abcam.com). Secondary anti-rabbit or anti-mouse IgG antibodies conjugated with peroxidase (1:2,000, Cell Signaling) were applied. Bands were visualized by enhanced chemiluminescence (ECL detection kit; Welgene, Daegu, Korea, http://www.jbilife.com). Physical protein binding of Nurr1^WT^ or Nurr1^Akt^ to candidate molecules (Akt, ubiquitin) and pAkt-mediated phosphorylation of Nurr1 proteins was determined using immunoprecipitation (IP) assays. NP cells transduced with Flag-tagged Nurr1^WT^ (Nurr1^Akt^) were harvested with RIPA buffer (50 mM HEPES, 150 mM NaCl, 1% NP40, 1 mM EDTA, 1 mM EGTA, 1 mM phenylmethylsulfonyl fluoride, 0.5% sodium deoxycholate, 1 mM Na_3_VO_4_) supplemented with protease inhibitors [11]. To examine ubiquitinylation of Nurr1 proteins, HEK293 cells were cotransfected with HA-tagged ubiquitin and Flag-Nurr1^WT^ (or Nurr1^Akt^). Cell lysates were incubated with anti-Flag antibody (Sigma: 5 μg/ml) for 2 hours in the cold room. After binding of antibodies to protein G beads for 2 h, the beads were washed three times with RIPA buffer and resuspended in sample buffer (Amresco, Cleveland, Ohio, http://www.amresco-inc.com/). Samples were electrophoresed through sodium dodecyl sulfate 10% polyacrylamide gels, transferred to nitrocellulose membranes, and probed by anti-HA, anti-Akt, or anti-pAkt substrate antibodies.

### Nurr1 Protein Stability

HEK293 cells were transfected with Nurr1^WT^ (or Nurr1^Akt^) and harvested during 6 hours of cycloheximide (100 μg/ml; Calbiochem) treatment. Nurr1 protein levels were determined by Western blot analyses.

### Reverse-Transcription Polymerase Chain Reaction

Standard reverse-transcription polymerase chain reaction (PCR) procedures were used. Optimal PCR conditions for each primer set were determined by varying MgCl_2_ concentrations, annealing temperatures, and cycle numbers to determine a linear amplification range. The primer sequences (forward and backward) and PCR conditions were as follows: GAPDH (5-GGCATTGCTCTCAATGACAA-3 and 5-AGGGCCTCTCTCTTGCTCTC-3, 25 cycles, 60°C, 165 bp); Nurr1 (5-TGAAGAGAGCGGACAAGGAGATC-3 and 5-TCTGGAGTTAAGAAATCGGAGCTG-3, 35 cycles, 57°C, 255 bp).

### Cell Toxicity Assays

NP cells were transduced with Nurr1^WT^ or Nurr1^Akt^ as described. After 6 days of in vitro differentiation, cell viability was determined in the cultures treated with H_2_O_2_ (50–500 uM; Sigma-Genosys) or 6-hydroxydopamine (6-OHDA; 50–200 uM; MP Biomedicals) for 8 hours by MTT assay (Sigma-Genosys), propidium iodide (PI) staining (Invitrogen), and by directly counting TH+ cells.

### In Vivo Transplantation

The 6-OHDA-lesioned rats were generated as described [12]. Neural precursor cells were harvested 2 days after Nurr1^WT^ or mutant transduction and dissociated into single cells in HBSS. Using a 22-gauge needle, 3 μl of cell suspension (1.5 × 10^5^ cells/μl in N2+bFGF) was deposited at the striatum (coordinates in AP, ML, and V relative to bregma and dura: -0.09, -0.42, -0.66 incisor bar set at 3.5 mm). The needle was left in place for 10 minutes following each injection. For histological analysis, animals were anesthetized with ketamine (4.5 mg/kg) mixed with rompun (93.28 μg/kg) and perfused transcardially with 4% paraformaldehyde in PBS. Brains were equilibrated with 30% sucrose in PBS and sliced on a freezing microtome (CM 1850, Leica). Free-floating brain sections (35-μm thick) were subjected to immunohistochemistry as described above. The total numbers of cells positive for Nurr1, TH, and DAPI in the graft were estimated by the Abercrombie correction factor [13].

### Cell Counting and Statistic Analysis

Cell counting was performed in microscopic fields randomly chosen (fractionator) across the culture area. Data are expressed as mean ± SEM of three independent experiments. Statistical comparisons were made by Student's *t* test or one-way analysis of variance (ANOVA) with post-hoc test using SPSS software (version 13.0; SPSS Inc., Chicago, IL, http://www.spss.com).

## RESULTS

### Degradation of Exogenous Nurr1 Proteins During In Vitro Precursor Differentiation

**Figure 1:**
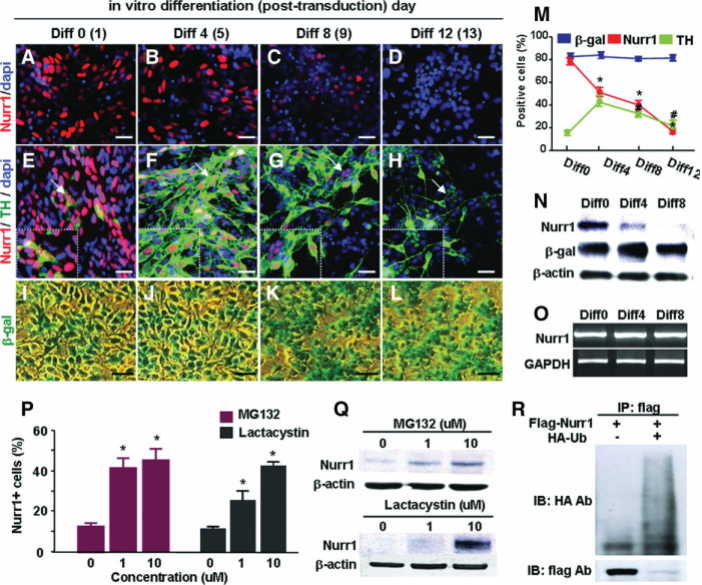
UPS-mediated protein degradation is responsible for the decrease in Nurr1+/TH+ cells during the differentiation of neural precursor cells in vitro. Neural precursor cells were cultured from nondopaminergic rat embryonic cortices at embryonic day 13, and transduced with Nurr1 or LacZ (control). On the day after transduction, differentiation of Nurr1-transduced precursors into DA neurons was induced for 12 days by withdrawing the mitogen bFGF. **(A–L):** Representative microscopic images for Nurr1+ **(A–D)** and Nurr1+/TH+ **(E–H)** cells from Nurr1-transduced cultures, and β-gal-stained cells **(I–L)** from LacZ-transduced cells over the in vitro differentiation period. Scale bar = 20 μm. Insets of **(E–H)** show enlarged views of the regions indicated by arrows. **(M):** Percent changes of Nurr1+, TH+, and β-gal+ cells from three independent cultures. Significant differences were found from the value of %Nurr1+ cells at differentiation day 0 (Diff0)* and from the %TH+ cells of Diff 4# (*p* < .01). Nurr1 protein **(N)** and mRNA **(O)** levels at Diff0, Diff3, and Diff8 were further determined.**(P–R):** UPS-mediated degradation of Nurr1 proteins. In the presence of the protein synthesis inhibitor cycloheximide (40 μg/ml), Nurr1+ cells **(P)** and Nurr1 protein levels **(Q)** were determined in the cortical precursor cells treated with the proteasome inhibitors MG132 or lactacystin (0, 1, and 10 μM) at differentiation day 6. **(R):** Immunoprecipitation assay for Ub and Nurr1 protein binding. *Significantly different from the untreated cultures (*p* < .01, *n* = 3 independent experiments).

Nurr1 expression was induced in cultured NP cells derived from rat fetal cortices. As demonstrated previously [10,14,15], exogenous Nurr1 expression yielded cells positive for TH, a key marker for DA neurons, from naïve nondopaminergic NP cells. Nurr1 immunoreactivity was localized in the nucleus of virtually all TH+ cells for up to 4 days of differentiation (Fig. 1F). The number of Nurr1-immunoreactive cells and levels of Nurr1 protein gradually decreased during the longer differentiation period (Fig. 1A–H, 1M, 1N) but without a significant change in Nurr1 mRNA levels (Fig. 1O), consequently yielding increased populations of TH+ cells which were negative for Nurr1 immunoreactivity (insets of Fig. 1G, 1H). In addition, reduced Nurr1 expression was followed by a substantial reduction in TH+ cell numbers after prolonged differentiation (Fig. 1F–H, 1M). As a control, protein levels of exogenous LacZ, expressed in a vector construct identical to that of Nurr1, were observed to be uniform and without variation throughout the cell differentiation period (Fig. 1I–L, 1M, 1N). Treatment of the cells with proteasome inhibitors MG132 or lactacystin significantly blocked Nurr1 protein degradation (Fig. 1P and 1Q). At differentiation day 6, Nurr1+ cells accounted for 15.2 ± 10.4% of total cells in untreated control versus 54.1 ± 10.6% in MG132 (10 μM)-treated cells (total 13,833 and 12,573 cells counted from three sets of independent cultures, *p* < 0.01, Student's *t* test). In an IP assay, Nurr1 proteins bound directly to ubiquitin (Ub) and slower migrating forms that corresponded to polyubiquitinylated species were visible (Fig. 1R). Leptomycin B, an irreversible inhibitor of CRM-1-dependent nuclear export [16], had no effect on Nurr1 decay (data not shown), suggesting degradation of Nurr1 in the nucleus.

### bFGF Prevents Nurr1 Protein Degradation

**Figure 2:**
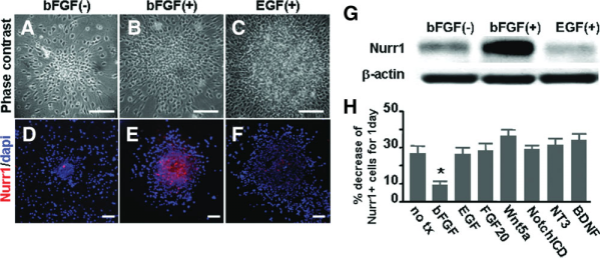
Basic fibroblast growth factor (bFGF) is specific to maintenance of Nurr1 protein stability. To maintain similar levels of cell-to-cell contact, which may influence Nurr1 protein stability, cortical precursors transduced with Nurr1 were cultured for 2 days in the form of floating cell aggregates (neurospheres) in the absence **(A,** **D)** or presences of the mitogens bFGF **(B, E)** or epidermal growth factor **(C, F)**, and then Nurr1 protein levels were determined **(G)**. Scale bars = 40 μm. In addition, neurospheres treated with various cytokines were plated on FN-coated surfaces and were stained against Nurr1. **(H):** Percent decreases of Nurr1+ cells for 1 day of in vitro culture. Nurr1-transduced precursor cells were left untreated (no tx) or treated with the factors and inhibitors indicated and the percent decreases were calculated by percent changes of Nurr1+ cell numbers before and 1 day after the treatments. *Significantly different from the untreated control (*p* < .01, *n* = 3, Student *t* test).

**Figure 3:**
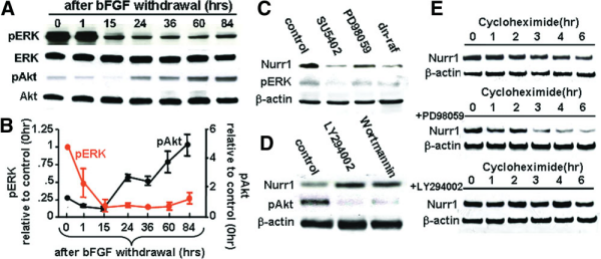
Raf-MEK and PI3K-Akt intracellular pathways downstream of basic fibroblast growth factor (bFGF) have opposing actions in the regulation of Nurr1 protein decay. **(A–C):** Opposing roles of extracellular regulated kinase (ERK) and Akt in the regulation of Nurr1 protein stability. **(A, B):** Time-course changes of ERK and Akt signal activations for 84 hours after bFGF withdrawal. The activated ERK and Akt levels were estimated by the ratios of pERK/ERK and pAkt/Akt, respectively. Each dot and bar in **(B)** represents the mean and SEM of the activated protein level (relative to that of time point 0) scanned from five Western blot analyses performed with three sets of independent cultures. **(C, D):** Nurr1 protein levels in the cultures treated with FGFR1 blocker (SU5402), Raf-ERK inhibitor (PD98059), dn-raf, or PI3K-Akt inhibitors (LY294002, wortmannin) were compared with the untreated control. **(E):** Effects of Raf-ERK and PI3K-Akt signals in Nurr1 protein stability. HEK-293 cells transfected with Nurr1 were treated with cycloheximide in the absence or presence of PD98059 or LY294002 and then harvested for a Western blot assay at the times indicated.

In contrast to exogenous Nurr1 protein decay during cell differentiation, the protein levels of Nurr1 in proliferating NP cells were maintained in the continued presence of bFGF in culture (Fig. 2D, 2E, 2G). These findings prompted us to determine if Nurr1 protein decay can be prevented by other mitogens acting on NP cells. Proliferation of NP cells was similarly induced by EGF treatment or activation of Notch signaling via Notch intracellular domain transduction (data not shown). However, neither of these factors was able to maintain the Nurr1 protein stability elicited by bFGF (Fig. 2D–H). Nurr1 protein levels were also not significantly altered by treatments with factors regulating NP cell differentiation (BDNF, NT3, FGF20, Wnt-5a) and survival (Fas ligand, pan-caspase inhibitor; Fig. 2H). The bFGF-sustained Nurr1 protein levels were abolished by treatment of cells with SU5402, an FGF receptor blocker (Fig. 3c). Together, these results suggest that the maintenance of Nurr1 proteins is specifically mediated by bFGF.

### Counteracting Regulatory Actions of Raf- and Akt-Mediated Intracellular Signals in Nurr1 Protein Stability

We next sought intracellular signals that act downstream of bFGF to maintain Nurr1 protein stability. To this end, we explored time-course changes of protein levels of activated (phosphorylated) forms of potential signaling molecules for the period after bFGF withdrawal. An immediate decrease of pERK levels was observed within 15 hours after bFGF withdrawal and pERK was present at reduced levels for the remainder of the differentiation period tested (Fig. 3A, 3B). In contrast, levels of pAkt, another potential signaling molecule downstream of bFGF [17,18], were slightly decreased during the initial period of bFGF withdrawal, but gradually and substantially increased for the rest of the differentiation period. The activation of Akt signaling was likely caused by the decrease in Raf/ERK activation, as the pAkt and Raf-ERK signals have been shown to mutually regulate each other in an inhibitory manner [19,20].

Inhibition of Raf-Erk signaling by the specific inhibitors PD98059 and U0126 or transduction of a dn-raf resulted in a marked reduction of Nurr1 protein levels (Fig. 3C and data not shown). On the contrary, the PI3K-Akt signal blockers LY294002 and wortmannin resulted in a striking increase of Nurr1 protein levels (Fig. 3D). To determine if the Nurr1 protein level changes were caused by Erk- or Akt-mediated regulation of protein degradation, we compared the stabilities of Nurr1 proteins in the absence and presence of inhibitors for these signaling molecules. Nurr1 proteins were readily degraded within 6 hours of cycloheximide treatment (Fig. 3E). Further drastic reduction of Nurr1 was observed to occur rapidly after PD98059 treatment. In contrast, Nurr1 protein levels in the cultures treated with LY294002 were stable after 6 hours of cycloheximide treatment, confirming counterregulatory roles of Raf-Erk and Akt signals in Nurr1 protein degradation.

### Direct Akt Phosphorylation Is Responsible for Nurr1 Ubiquitylation

**Figure 4:**
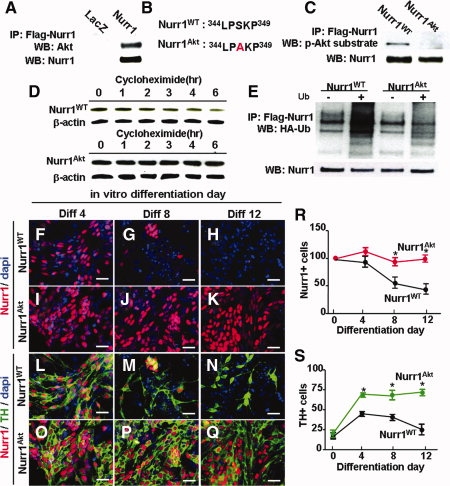
Enhanced maintenance of TH+ DA cells generated by Nurr1^Akt^ transduction. **(A):** Direct protein binding of Akt to Nurr1. Cortical neural precursor (NP) cells transduced with Flag-Nurr1 were immunoprecipitated by incubation with Flag antibody and immunoblotted with Akt antibody. **(B):** Sequence alignment of Nurr1 proteins around the consensus Akt phosphorylation site. **(C):** Abolishment of Akt phosphorylation in Nurr1^Akt^ mutant proteins in which serine 347 is transformed into alanine. Rat cortical NP cells transduced with Flag-Nurr1^WT^ or Flag-Nurr1^Akt^ were harvested, immunoprecipitated with Flag antibody, and then immunoblotted by phospho-(Ser/Thr) Akt substrate antibody. **(D):** Abolishment of the Akt phosphorylation site in Nurr1 enhances protein stability. Nurr1 protein levels were compared in the cultures transfected by Nurr1^WT^ and Nurr1^Akt^ during 6 hours of cycloheximide (100 μg/ml) treatment. **(E):** Decreased ubiquitinylation in the Nurr1 Akt phosphorylation mutant. Immunoprecipitation assays for Ub and Nurr1 protein binding were performed as described in Figure 1. **(F–S):** Maintenance of Nurr1+/TH+ DA neurons in cell cultures transduced with Nurr1^Akt^. **(F–Q):** Representative microscopic images for Nurr1+ **(F–K)** and Nurr1+/TH+ **(L–Q)** cells from the cultures transduced with Nurr1^WT^ and Nurr1^Akt^ over the differentiation period in vitro. Scale bar = 20 μm. Percent Nurr1+ and TH+ cells are depicted in **(R)** and **(S)**, respectively. *Significantly different from the respective Nurr1^WT^ values for the same differentiation days (*p* < .01).

We next investigated the possibility that Raf-Erk and Akt molecules function through direct interaction with Nurr1 proteins. Direct protein interactions of Akt (Fig. 4A) and Erk1/2/5 with Nurr1 were observed in IP assays [21,22] (data not shown). Differentiation-dependent decreases of a Nurr1 mutant protein, in which all three Erk phosphorylation consensus sites were abolished, was comparable and insignificantly different from that of wild-type Nurr1 (data not shown), ruling out the possibility of direct Erk phosphorylation of Nurr1 mediating the maintenance effect. The Nurr1 protein contains a consensus site for Akt phosphorylation at Ser 347 (Fig. 4B). As shown in Figure 4C, pAkt substrate antibody, which recognizes phosphorylated peptides and proteins at the Akt target motif (RXRXXS/T), readily binds to Nurr1^WT^, but not to the Nurr1^Akt^ in which serine 347 is substituted by alanine, indicating Akt-mediated phosphorylation of Nurr1 and abolishment of this Akt-mediated phosphorylation in the mutant.

The effect of the mutation was dramatic, with a clear difference in the protein stability of Nurr1^Akt^ compared to that of Nurr1^WT^ after cycloheximide treatment (Fig. 4D). Nurr1 ubiquitylation was significantly reduced in Nurr1^Akt^-transfected cells, indicating that the effect of the Akt mutation is elicited by preventing initiation of UPS-mediated Nurr1 protein degradation (Fig. 4E).

### Phenotype Maintenance and Cell Survival of TH+ DA Cells Generated by Nurr1^Akt^ Transduction

Consistent with the sustained protein stability of Nurr1^Akt^, no significant decreases in the percentage of Nurr1+ cells were seen in the cortical precursor cells transduced with Nurr1^Akt^ during 12 days of differentiation in vitro (percent Nurr1+ cells out of total cells: 67.5 ± 1.8% (Diff0), 69.2 ± 4.0% (Diff4), 74.5 ± 12.8% (Diff8), and 63.9 ± 1.4% (Diff12); *p* = 1.0 compared to Diff0 and Diff12, *n* = 3 sets of independent cultures, one-way ANOVA with post-hoc test, total 6,012–13,260 cells counted), whereas a greater than 60% loss in the percentage of Nurr1+ cells was observed during the same period of time in Nurr1^WT^-transduced cultures (Fig. 4F–K). We further confirmed that the effect of the Akt mutation is not likely due to enhanced transcription of the Nurr1 gene, as mRNA levels of Nurr1^Akt^ and Nurr1^WT^ were indistinguishable (data not shown). Along with the sustained Nurr1 protein levels, TH+ cell numbers in the Nurr1^Akt^-transduced cultures were stably maintained during the in vitro differentiation period. For instance, the percentages of TH+ cells were 66.5 ± 2.8% at Diff4, 60.3 ± 7.4% at Diff8, and 71.5 ± 2.0% at Diff 12 (total 7,005, 11,985, and 13,260 cells counted, respectively, from three independent experiments, *p* = .873 compared to Diff0 and Diff12 by ANOVA with post-hoc Bonferroni test) in the Nurr1^Akt^-transduced cultures (Fig. 4L–Q, 4S).

**Figure 5:**
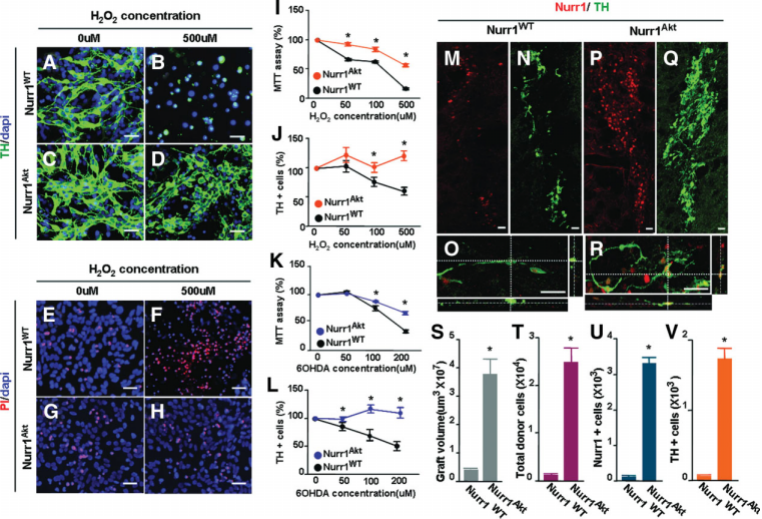
Nurr1^Akt^-transduced NP cells yield dopamine (DA) neurons that are resistant to toxic stimuli and show enhanced survival in vitro and in vivo after transplantation. **(A–L):** Cellular toxicity induced by H_2_O_2_ (50–500 μM) or 6-OHDA (50–200 uM) treatments. After 6 days of in vitro differentiation, cells were treated with the toxins for 8 hours, and cell viability was determined by the MTT assay **(I, K)**, PI staining **(E–H)**, and directly counting TH+ cells **(J, L)**. Scale bar = 20 μm. *Significantly different from the respective Nurr1^WT^ values of the same toxin concentrations (*p* < .01, *n* = 3 independent experiments). **(A–D)** and **(E–H)** are the representative images of TH+ cells and PI-stained cells, respectively, which demonstrate the difference in H_2_O_2_-induced cell toxicity in Nurr1^WT^- and Nurr1^Akt^-transduced cultures. **(M-V):** In vivo survival of TH+/Nurr1+ cells after transplantation. Representative images for TH+ **(N, Q)**, Nurr1+ **(M, P)**, and TH+/Nurr1+ **(O, R)** cells in the grafts generated by Nurr1^WT-^ **(M–O)** and Nurr1^Akt-^ **(P–R)** transduced precursors. Quantification of graft volumes **(S)**, total donor cells **(T)**, Nurr1+ **(U)**, and TH+ **(V)** cell numbers in the grafts are shown. Scale bar = 20 μm. *Significantly different from the respective Nurr1^WT^ values (*p* < .01, *n* = 5 for each value, Student's *t* test).

Messenger RNA expression of DA phenotype genes such as TH and DA transporter were increased in Nurr1^Akt^-transduced cultures compared to control cultures (data not shown). Maintenance of the DA phenotype in Nurr1^Akt^-transduced cultures is likely to be achieved by the continuous transcriptional activation resulting from sustained levels of Nurr1^Akt^ proteins. In addition, Nurr1 may have a cell survival effect in TH+ cells because Nurr1 has been shown to act as a cell survival factor [2,4,5]. Cell apoptosis was markedly decreased in Nurr1^Akt^-transduced cultures than in those with Nurr1^WT^ (percent cells with apoptotic nuclei: 2.7 ± 0.7% in Nurr1^Akt^ vs. 13.2 ± 1.3% in Nurr1^WT^-transduced cultures at Diff6, total 11,397 and 11,462 cells counted, *n* = 3, *p* < .01). Furthermore, Nurr1^Akt^-transduced cells were more resistant to the cellular toxicity induced by H_2_O_2_ and 6-OHDA treatments based on our estimation of cell viability using the MTT assay (Fig. 5I, 5K), the percentage of TH+ cells (Fig. 5J, 5L), and the PI staining (Fig. 5E, 5H, data not shown). Thus, sustained Nurr1 protein stability in Nurr1^Akt^-transduced precursor cells preserves DA phenotypes and improves cell survival. These events contribute in turn to the maintenance of TH+ cells during differentiation.

Finally, we examined the in vivo survival effect of Nurr1^Akt^ expression in donor precursor cells after transplantation into the striatum of 6-OHDA-lesioned rats. Two weeks after transplantation, none or very few donor cells were viable in the striatum grafted with Nurr1^WT^-transduced NP cells (Fig. 5M, 5N). Furthermore, most of the TH+ cells detected in the striatum of the animals grafted with Nurr1^WT^-cells were negative for Nurr1 (Fig. 5O). In contrast, Nurr1^Akt^-transduced precursors survived, integrated into host striatum to generate tubular masses of grafts, and differentiated toward TH+ cells (Figs. 5P, 5Q), most of which expressed Nurr1 proteins (Fig. 5R). Graft volumes (Fig. 5S), total donor cells (Fig. 5T), Nurr1+ cells (Fig. 5U), and TH+ cell numbers per graft (Fig. 5V) were much greater in the animals grafted with Nurr1^Akt^-transduced precursors (Fig. 5). For instance, TH+ cells per graft were 1,757.5+155.4 (Nurr1^Akt^) versus 53.12+20 (Nurr1^WT^), *n* = 5, *p* < .01). Altogether, our findings indicate that Nurr1^Akt^ overexpression in donor cells improves cell survival and DA phenotype maintenance in transplanted cells.

## DISCUSSION

In this report, we showed that Nurr1 proteins undergo UPS-mediated degradation during precursor cell differentiation and that the process is regulated by the opposing actions of ERK and Akt intracellular signals. We further demonstrated that direct Akt phosphorylation of Nurr1 protein controls the ubiquitinylation of this protein and that stability of Nurr1 proteins can be sustained by abolishing the Akt phosphorylation site of Nurr1. These findings are novel and are supported by previous studies exemplified as follows. It is thought that protein phosphorylation plays a critical role in the initiation of protein ubiquitylation [23–25]. ERK and Akt have recently been specified as the critical protein phosphorylation pathways controlling several protein degradations [26–28]. In support of our findings, opposing cellular responses mediated by Raf and Akt signals have also been shown to control UPS-degradation of p53 proteins [29]. Furthermore, similar to our results, direct Akt-mediated phosphorylation of androgen receptor, a member of the same nuclear-hormone receptor family of proteins that Nurr1 belongs to, activates ubiquitinylation of this protein [28]. Current knowledge supports the idea that the nucleus is the primary target for degradation of nuclear receptors [30]. Consistent with this theory, treatment with the nuclear export inhibitor, leptomycin B, did not affect Nurr1 degradation, indicating localization of Nurr1-specific UPS-mediated degradation to the nucleus. Further molecular understanding of Nurr1 degradation requires studies to define Nurr1-specific E3 ligases and deubiquitinylation enzymes.

The most critical problem with current methods of cell transplantation for PD treatment is the low viability of donor cells. Only a minor portion of the DA neurons survive transplantation [8,31]. Furthermore, recent studies have demonstrated that the diseased PD environment transmits a toxic signal to the grafted neurons [32], indicating that improvement of host environment will also be required to improve the survival of grafted neurons. Fundamental corrections to the environment created by the diseased state, however, do not seem to be easily achievable. An alternative would be to provide transplanted cells with resistance to the toxic environment. In addition to the key issue of survival, unstable phenotypes of transplanted DA neurons [9] also contribute to a low yield of DA neurons after transplantation. The present study shows an example of genetic manipulation yielding donor DA cells with improved cell survival and better-maintained phenotypes in vitro and in vivo after transplantation. The single mutation of the Akt-phosphorylation site of Nurr1 in this study resulted in a dramatic effect on Nurr1 protein stability, and in turn maintenance of TH+ cells. It is manifest that the continued Nurr1 expression in the Nurr1^Akt^-transduced cells accounts for the observed effect of DA phenotype maintenance, as Nurr1 is a transcription factor that activates expression of genes involved in the DA phenotype [1,2,6]. The majority of the TH+ cells derived from Nurr1^Akt^-transduced precursors expressed Nurr1 for a prolonged period during differentiation. Consistent with a previous study [33], the Nurr1-expressing DA cells maintained their DA phenotype better and survived longer. Cell transplantation in clinical level, however, requires long period of donor cell survival. While enhanced Nurr1+/TH+ cell yield was clear and striking by Nurr1^Akt^-transduced cell transplantation at 2 weeks after transplantation (Fig. 5M–V), the beneficial effect of the Nurr1 mutation was not continued for a longer period after transplantation: only few TH+ cells (less than 200 TH+ cells/graft) were detected in the brains grafted with Nurr1^Akt^-cells 8 weeks after transplantation. We found that lack of the long-term effect is mainly due to unstable exogene (Nurr1) mRNA expression, but not due to loss of the Akt mutation effect in maintaining Nurr1 protein stability in the transplanted brains in vivo (data not shown). This is consistent to the previous studies demonstrating loss of exogene (GFP) expression in donor cell after neural transplantation [34,35]. We further found that promoters of expression vectors (LTR, CMV, EF1α) commonly contain cAMP-response element (CRE) and the promoter-driven expressions are highly dependent on activated CRE-binding protein (CREB) intracellular signal (data not shown). Thus inactivation of CREB signal in donor cells long after transplantation, with unidentified mechanism, is likely to be responsible for loss of Nurr1 mRNA expression and in turn loss of Nurr1-induced TH phenotype expression in those cells. We are now investigating to develop methods to achieve stable exogene expression in transplanted donor cells.

DA neurons yielded by the mutant Nurr1 were more resistant to toxic stimuli. These findings indicate an advance in cell therapeutic approaches for PD through generation of donor DA cells that are able to sustain their phenotype after transplantation and that survive by overcoming the pathologic host environment of PD. Nurr1 is a susceptible factor in PD and decreased levels of Nurr1 have been found in PD patients [7], suggesting that Nurr1 is a target molecule for the treatment and prevention of PD. We anticipate that the information we presented in this study regarding Nurr1 protein degradation and stability can be used to develop medical treatments to prevent the progression of PD.

## DISCLOSURE OF POTENTIAL CONFLICTS OF INTEREST

The authors indicate no potential conflicts of interest.
